# CNV-TV: A robust method to discover copy number variation from short sequencing reads

**DOI:** 10.1186/1471-2105-14-150

**Published:** 2013-05-02

**Authors:** Junbo Duan, Ji-Gang Zhang, Hong-Wen Deng, Yu-Ping Wang

**Affiliations:** 1 Department of Biomedical Engineering, Tulane University, New Orleans, USA; 2 Department of Biostatistics and Bioinformatics, Tulane University, New Orleans, USA; 3 Center for Bioinformatics and Genomics, Tulane University, New Orleans, USA

## Abstract

**Background:**

Copy number variation (CNV) is an important structural variation (SV) in human genome. Various studies have shown that CNVs are associated with complex diseases. Traditional CNV detection methods such as fluorescence *in situ* hybridization (FISH) and array comparative genomic hybridization (aCGH) suffer from low resolution. The next generation sequencing (NGS) technique promises a higher resolution detection of CNVs and several methods were recently proposed for realizing such a promise. However, the performances of these methods are not robust under some conditions, *e.g.*, some of them may fail to detect CNVs of short sizes. There has been a strong demand for reliable detection of CNVs from high resolution NGS data.

**Results:**

A novel and robust method to detect CNV from short sequencing reads is proposed in this study. The detection of CNV is modeled as a change-point detection from the read depth (RD) signal derived from the NGS, which is fitted with a total variation (TV) penalized least squares model. The performance (*e.g.*, sensitivity and specificity) of the proposed approach are evaluated by comparison with several recently published methods on both simulated and real data from the 1000 Genomes Project.

**Conclusion:**

The experimental results showed that both the true positive rate and false positive rate of the proposed detection method do not change significantly for CNVs with different copy numbers and lengthes, when compared with several existing methods. Therefore, our proposed approach results in a more reliable detection of CNVs than the existing methods.

## Background

Copy number variation (CNV) [1] has been discovered widely in human and other mammal genomes. It was reported that CNVs are present in human populations with high frequency (more than 10 percent) [2]. Various studies showed that CNVs are associated with Mendelian diseases or complex diseases such as autism [3], schizophrenia [4], cancer [5], Alzheimer disease [6], osteoporosis [7], *etc.*

CNV is commonly referred to as a type of structural variations (SVs), and involves a duplication or deletion of DNA segment of size more than 1 kbp [8]. The mechanism by which CNVs convey with phenotypes is still under study. A widely accepted explanation is that, if a CNV region harbors a dosage-sensitive segment, the gene expression level varies, which leads to the abnormality of related phenotype consequently [9].

Before the emergence of next generation sequencing (NGS) technologies, methods such as fluorescence *in situ* hybridization (FISH) and array comparative genomic hybridization (aCGH) were employed to detect CNVs. The main problem of these methods is their relatively low resolutions (about 5 ∼10 Mbp for FISH, and 10 ∼25 kbp with 1 million probes for aCGH [10]). With the rapid decrease of the cost of NGS, high coverage sequencing became feasible, offering high resolution CNV detection. After Korbel *et al.*’s work of detecting CNVs from NGS data [11,12], many CNV detection methods have been developed recently [10,13-23]. However, as shown in our previous study [24], the performances of the existing methods are not robust; *e*.g., CNVnator degenerates at small single copy length; and readDepth degenerates at low copy number variation (see the simulation). So new methods are needed for reliable detection of CNVs.

Methodologically, there are mainly two ways to detect CNVs from NGS data [25]: pair-end mapping (PEM) and depth of coverage (DOC) based methods. The PEM based method is commonly used to detect insertion, deletion, inversion, *etc.*[26]. After the pair ends from the test genome being aligned to the reference genome, the span between the pair ends of the test genome is compared with that of the reference genome. The significant difference between the two spans implies the presence of a deletion or insertion event. There are several DOC based methods, such as CNV-seq [14], FREEC [20], readDepth [21], CNVnator [22], SegSeq [13], and event-wise testing (EWT) [10]. The principle of DOC based methods is: the short reads are randomly sampled on the genome, so when the short reads are aligned to the reference genome, the density of the short reads is locally proportional to the copy number [10]. Based on the probability distribution of the read depth (RD) signal, a statistical hypothesis testing will tell whether a CNV exists or not. Specifically, the procedure of DOC based methods include: aligned reads are first piled up and then the read counts are calculated across a sliding [14] or non-overlapping windows (or bins) [10,13,20,22], yielding the so-called RD signal. The ratio of the read counts (case *vs.* matched control) is used by CNV-seq [14] and SegSeq [13], so further normalization is not required [18]. Otherwise, normalization such as GC-content [10,22] and mapability [21] correction is required. The normalized read depth signal (or the raio) is analyzed with either of the following procedures: (1) segmented or partitioned by change-point detection algorithms, and followed with a merge procedure [13] (*e.g.* readDepth [21] and CNVnator [22] utilize circular binary segmentation (CBS) and mean shift, respectively). (2) tested by a statistical hypothesis at each window (*e.g.*, event-wise testing (EWT) [10]) or several consecutive windows (*e.g.*, CNV-seq [14]).

We propose a total variation (TV) penalized least squares model to fit the RD signal, based on which the CNVs are detected with a statistical testing. We name the method as the CNV-TV. CNV-TV assumes that a plateau/basin in the RD signal correspond to a duplication/deletion event (*i.e.*, CNV). Then a piecewise constant function is used to fit the RD signal with the TV penalized least squares, from which the CNVs are detected. It is often cumbersome to determine the tuning of the penalty parameter in the model, which controls the tradeoff between sensitivity and specificity. Therefore, the Schwarz information criterion (SIC) [27] is introduced to find the optimal parameter. The proposed method may be applied either to paired data (tumor *v.s.* control in oncogenomic research) or to single sample that has been adjusted for technical factors such as GC-content bias. The key feature of the CNV-TV method is its robust performance, *i.e.,* the detection sensitivity and specificity keeps stable when detecting CNVs with short length or near-normal copy number. Compared with several recently published CNV detection methods on both simulated and real data, the results show that CNV-TV can provide more robust and reliable detection of CNVs.

## Methods

The first step to process the raw NGS data is to align (or map) the short reads with a reference genome (or template, NCBI37/hg19, for example) by alignment tools such as MAQ [28] and Bowtie [29]. Then the aligned reads are piled up, and read depth signal *y*_*i*_,(*i*=1,2,…,*n*) is calculated to measure the density of the aligned reads, where *n* is the length of the read depth signal. There are several ways to calculate *y*_*i*_, for example, Yoon *et al.*[10] used the count of aligned reads that fall in a non-overlapping window with size 100 bp, while Xie and Tammi [14] used a sliding window with 50% overlap.

**Figure 1:**
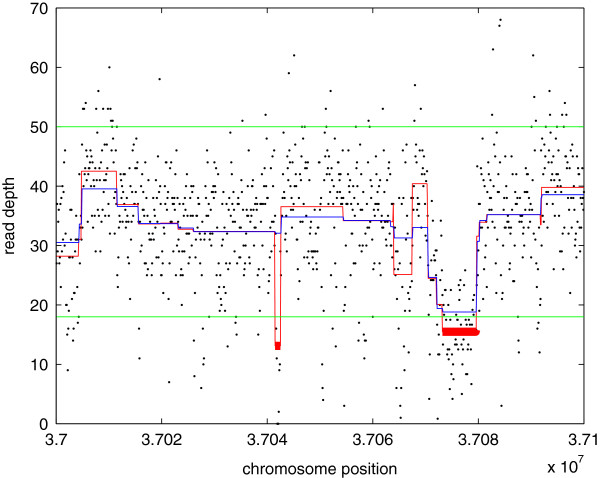
**The processing result of the region chr21:37.0∼37.1 Mbs (zoom in of the region between the vertical magenta lines in Figure **6). The black dots are the read depths; the blue line is the smoothed signal *x*_*i*_; the red line is the corrected smoothed signal ${\tilde{x}}_{i}$; the horizontal green lines are the lower and upper cutoff values estimated from the histogram; and the thick red lines highlight the detected CNVs. Note that a small CNV at region 37.04 with length 1.1 kbp is detected.

The detection of CNVs from read depth signal *y*_*i*_ can be viewed as a change-point detection problem (see Figure 1 where *y*_*i*_’s are the black dots). There exist many methods to address this problem [30]. The total variation (TV) based regularization method has been widely used in the signal processing community to remove noise from signals [31]. In this paper, we use the total variation penalized least squares as shown in Eq. (1) to fit the RD profile, based on which a statistical test is used to detect CNVs.

(1) $$\begin{array}{lcrr} \underset{x_{i}}{\min}\left\{\frac{1}{2}\overset{n}{\underset{i=1}{\sum }}{\left(y_{i}-x_{i}\right)}^{2}+\lambda \overset{n-1}{\underset{i=1}{\sum }}\phi (x_{i+1}-x_{i})\right\}. & & & \end{array}$$

In Eq. (1), the first term is the fitting error between *y*_*i*_ and the recovered smooth signal *x*_*i*_; the second term is the total variation penalty: when a change-point presents between *x*_*i*_ and *x*_*i*+1_, a penalty *ϕ*(*x*_*i*+1_−*x*_*i*_) is imposed. The penalty function *ϕ*(*x*) is usually a symmetric function that is null at the origin and monotonically increases for positive *x*. The ideal choice of *ϕ*(*x*) is the *ℓ*-0 norm of *x*. However the *ℓ*-0 norm yields an NP-hard problem, which is computationally prohibitive. Instead, convex or non-convex relaxations of *ℓ*-0 norm are of greater interest, such as Huber function [32], truncated quadratic [33]*etc.* In recent compressed sensing theory [34,35], *ℓ*-1 norm penalized models [36] received wide attention because of their robust performance, as well as the availability of fast algorithms such as the homotopy [37,38] and least angle regression (LARS) [39]. For these reasons, we select the *ℓ*-1 norm as the penalty function *ϕ*(*x*).

*λ* is the penalty parameter, which controls the tradeoff between the fitting fidelity (or fitting error) and penalty caused by the change-points. When *λ*→0, the effect of penalty term is ignorable and the solution is *x*_*i*_=*y*_*i*_. On the contrary, when *λ*→+*∞*, the effect of fitting fidelity term is ignorable and the solution is $x_{1}=x_{2}=\ldots =x_{n}={\bar{y}}_{i}$, indicating that there is no change-point (here ${\bar{y}}_{i}$ is the mean of *y*_*i*_). As a result, when *λ* decreases from +*∞* to 0, the change-points can be detected one by one according to their significance level. The notation *x*_*i*_(*λ*),(*i*=1,2,…,*n*), which characterizes the evolution of solution *x*_*i*_ with respect to *λ*, is termed as the set of solutions.

To simplify notations in Eq. (1) for further presentation, ***y*** and ***x*** are introduced as the the vector forms of *y*_*i*_ and *x*_*i*_ respectively, *i.e.****y***=[*y*_1_,*y*_2_,…,*y*_*n*_]^*T*^, and ***x***=[*x*_1_,*x*_2_,…,*x*_*n*_]^*T*^, where *T* represents the transpose operation. Therefore, the matrix form of Eq. (1) reads:

(2) $$\begin{array}{lcrr} \underset{x}{\min}\left\{\frac{1}{2}∥y-x{∥}^{2}+\lambda ∥\mathrm{Dx}{∥}_{1}\right\}. & & & \end{array}$$

where ∥·∥^2^ is the sum of squares of a vector; ∥·∥_1_ denotes the *ℓ*-1 norm, *i.e.* the sum of absolute values of each entry in a vector; and ***D*** is a matrix of size (*n*−1)×*n* that calculates the first order derivatives of signal ***x*** (note that the first entry of ***Dx*** is *x*_2_−*x*_1_, the second is *x*_3_−*x*_2_, *etc.*):

(3) $$\begin{array}{lcrr} D=\left[\begin{array}{ccccc} -1 & 1 & 0 & \cdots & 0\\ 0 & -1 & 1 & \ddots & \vdots \\ \vdots & \ddots & \ddots & \ddots & 0\\ 0 & \cdots & 0 & -1 & 1 \end{array}\right]. & & & \end{array}$$

Harchaoui and Lévy-Leduc [40] proposed to use the LASSO [41] to solve an alternative form of Eq. (2). In [42] we presented an algorithm to estimate directly the set of solutions of Eq. (2). In fact, Eq. (2) is equivalent to the following problem [43]:

(4) $$\begin{array}{lcrr} \underset{u}{\min}\left\{\frac{1}{2}∥z-\mathrm{Au}{∥}^{2}+\lambda ∥u{∥}_{1}\right\}, & & & \end{array}$$

where

(5) $$\begin{array}{l} \begin{array}{cc} z= & D^{T}{\left({\mathrm{DD}}^{T}\right)}^{-1}\mathrm{Dy}\\ A= & D^{T}{\left({\mathrm{DD}}^{T}\right)}^{-1}\\ u= & \mathrm{Dx} \end{array} \end{array}$$

Eq. (4) is the *ℓ*-1 norm based regression, and thus can be solved efficiently using algorithms like homotopy [37,38] and least angle regression (LARS) [39]. Once ***u*** is known, ***x*** can be obtained as [44]

(6) $$\begin{array}{lcrr} x=y+D^{T}{\left({\mathrm{DD}}^{T}\right)}^{-1}(u-\mathrm{Dy}). & & & \end{array}$$

As mentioned previously, both the robust performance and the availability of efficient numerical algorithms are our considerations for choosing the *ℓ*-1 norm based penalization. Another attracting property of *ℓ*-1 norm is that it yields sparse solution [45], *i.e.,****u*** is a sparse vector with a limited number of non-zero values. Consequently, ***x***, the first order integral of ***u***, is a piece-wise constant signal, which is our basic assumption about the read depth signal.

If the set of solutions {*x*_*i*_(*λ*_*k*_)|*i*=1,2,…,*n*;*k*=1,2,…,*K*} of Eq. (2) is known, change-points can be sorted according to their significance by tuning *λ* from *λ*_1_=+*∞* to *λ*_*K*_=0. Here *K* is the number of transition points of the solution when *λ* decreases from +*∞* to 0 [46], which can be estimated by a LASSO solver.

A user can make the final decision on which *λ* to use. However, an automatic approach to choose this parameter is desirable. In the following, the model selection technique is employed to address this problem. In our problem, the degree of the model is the number of pieces in the smoothed read depth signal *x*_*i*_, or the number of change-points plus one. A few commonly used model selection methods include *L*-curve [47], Akaike information criterion [48], Schwarz information criterion (SIC) [27], *etc.* Here, the SIC is adopted because of its robust performance [49], and has been used in our earlier study for detecting CNVs from aCGH data [50].

**Figure 2:**
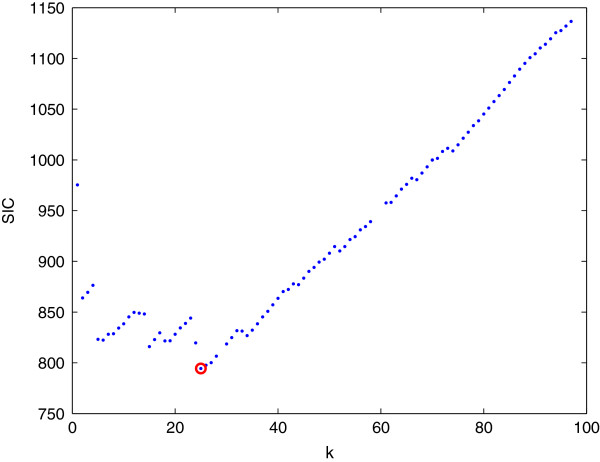
**The SIC curve of Figure **1. Each blue dot corresponds to solution with *SIC*(*λ*_*k*_). The red circle is the minimum, which corresponds to the optimal solution ${\tilde{x}}_{i}(\hat{\lambda })$.

Since the *ℓ*-1 norm based solution is biased [51], a correction is needed first. For solutions *x*_*i*_(*λ*_*k*_)^′^*s*,(*i*=1,2,…,*n*) at *λ*_*k*_, first they are segmented into pieces such that within the piece I={i,i+1,…i+l}, *x*_*i*_=*x*_*i*+1_=…=*x*_*i*+*l*_ (here we omit the dependency on *λ*_*k*_), and at change-points *x*_*i*−1_≠*x*_*i*_,*x*_*i*+*l*_≠*x*_*i*+*l*+1_. Then the correction is carried out piece by piece. For each piece I, the mean of *y*_*i*_ within this piece is used as the amplitude of *x*_*i*_, *i.e.*, ${\tilde{x}}_{i}={\tilde{x}}_{i+1}=\ldots ={\tilde{x}}_{i+l}=\frac{\overset{i+l}{\underset{i}{\sum }}y_{i}}{l+1}$ (see Figure 1, where *x*_*i*_ is the blue line and ${\tilde{x}}_{i}$ is the red one). The SIC at *λ*_*k*_ is calculated as:

(7) $$\begin{array}{lcrr} \text{SIC}(\lambda_{k})=m\ln(n)+\frac{\overset{n}{\underset{i=1}{\sum }}{\left(y_{i}-{\tilde{x}}_{i}\right)}^{2}}{\sigma^{2}}, & & & \end{array}$$

where *m* is the number of pieces, and *σ*^2^ is the variance of noise, which can be estimated manually from the region that does not harbor any CNV. The optimal *λ* is achieved at (see Figure 2):

(8) $$\begin{array}{lcrr} \hat{\lambda }=\arg\underset{k}{\min}\text{SIC}(\lambda_{k}). & & & \end{array}$$

Once $\hat{\lambda }$ is known, the optimal smooth signal of *y*_*i*_ is ${\tilde{x}}_{i}(\hat{\lambda })$; then a CNV can be identified as a segment with significantly abnormal amplitude, *i.e.* the amplitude below or above some predefined cutoff values. This cutoff values can either be estimated from the noise variance, or be estimated adaptively from the histogram of the read depth signal since the distribution of the read depth signal can be modeled as a mixture of Poisson distributions [52]. After the region of CNV is estimated, the copy number value can be estimated as the ratio between the reads count of the CNV region in the test genome and that of the corresponding region in the reference or control genome.

## Results

We evaluated the proposed method on both simulated and real data, and compared the results with six representative CNV detection methods.

A number of CNV detection methods have been published recently for NGS data analysis [10,13-23], and these methods are different in the use of statistical model, parameter, methodology, programming language, operating system, input requirement, output format, *etc.*; a comparative study of these different methods has been conducted by us [24]. Based on these factors, as well as the availability and the citation of these methods in literatures, six popular and representative methods were selected: CNV-seq [14], FREEC [20], readDepth [21], CNVnator [22], SegSeq [13], and event-wise testing (EWT) [10].

The parameters of selected CNV detection methods were tuned to achieve their best performances in the sense that their sensitivities are maximized while the false positive rates are controlled below 1e-3. The criteria of tuning the parameters are given as follows: (1) the shared parameters are set the same for fairness. For example, the thresholds for CNV-seq and FREEC are set to 0.6; the *p*-values of CNV-seq, *P*_*init*_ and *P*_*merge*_ of SegSeq, false detection rate of readDepth are set to 1e-3; the bin size of CNVnator is set to 100 bp since the recommended bin size of GC-content correction is 100 bp for both readDepth and EWT. The smallest *H*_*b*_ parameter (number of consecutive bins) of CNVnator is 8, so the ‘filter’ parameter of EWT is also set to 8. With this parameter, the smallest detectable CNV has the length of 800 bp, so the window size of FREEC and SegSeq is set to 800 bp. (2) The unique parameter of each method is tested after the shared parameters are fixed. In summary, the parameters are as follows: for CNV-seq, ‘p-value’ is set to 1e-3, and ‘log2-threshold’ is set 0.6; the ‘bin_size’ of CNVnator is set to 100 bp. For readDepth, ‘fdr’ is set to 1e-3; ‘overDispersion’ is set to 1; ‘readLength’ is set to 36 bp; ‘percCNGain’ and ‘percCNLoss’ are set to 0.01; ’chunkSize’ is set to 5e6. For EWT, the bin size ‘win’ is set to 100 bp; and ‘filter’ is set to 8. For SegSeq, the window size is set to 800 bp; the break-point *p*-value ‘p_bkp’ and merge *p*-value ‘p_merge’ are set to 1e-3. For FREEC, ‘window’ is set to 800 bp; ‘step’ is set to 400 bp; and the threshold is set to 0.6. Parameters not mentioned here are set to default.

For CNV-TV, the read depth signal was calculated from the BAM file with SAMtools [53], with the window size of 100 bp. The GC-content bias [54] was corrected using the profile file of RDXplorer [10]. The corrected read depth signal was then segmented by the proposed method. The matlab function *SolveLasso* from the SparseLab package (http://sparselab.stanford.edu/) was used to estimate the set of solutions of Problem (4). The noise variance *σ* in Eq. 7 was calculated as the median of the standard deviations of 10 segments with length 10 kbp, which are evenly distributed on the whole chromosome. The cutoff value to call a CNV was determined by the histogram of the corrected read depth signal, such that both the left and right tail areas cover five percent of the whole distribution.

### Simulated data processing

**Figure 3:**
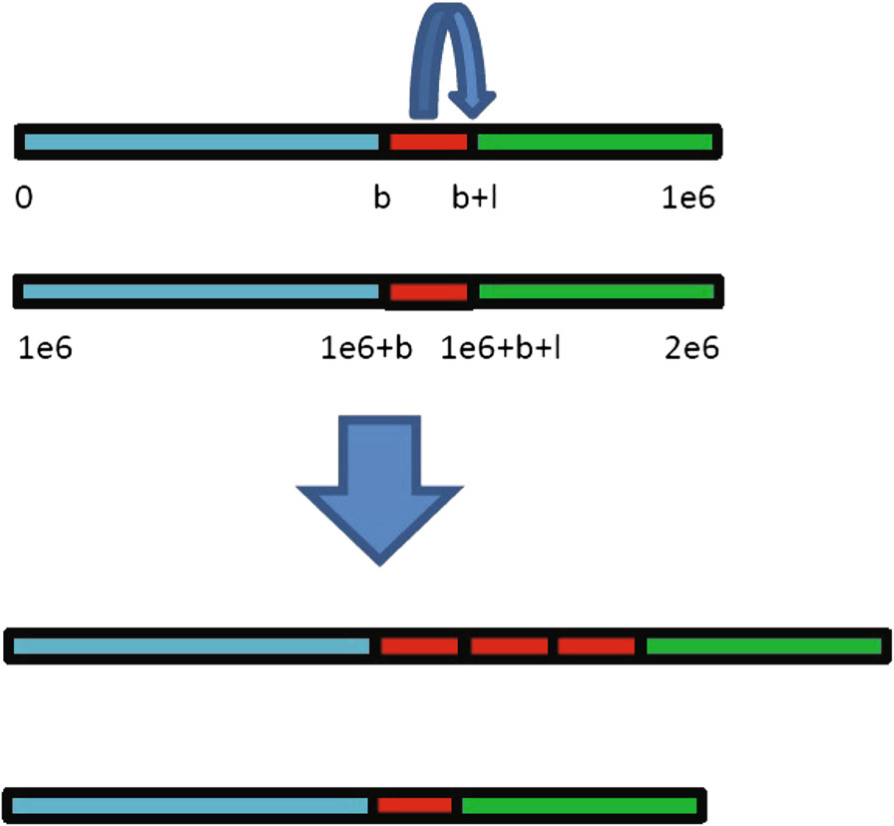
**A schematic demonstration of the generation of test genome (the lower figure) from the reference genome (the upper one) in the simulation study.** A DNA section of single copy length *l* bp (the length of a single red block) starting from genomic locus *b* is copied and inserted *c*−2 times. In the displayed test genome (the lower), the copy number *c* (the number of red blocks) is 4.

To test the performance of CNV-TV comprehensively for a set of conditions (copy number *c* and single copy length *l*), simulations were carried out. 1000 Monte Carlo trials were run repeatedly for each condition. In the first experiment, the effect of single copy length (the length of red block in Figure 3) was tested, which changes from 1 kbp to 6 kbp. In the second experiment, the effect of copy number (the number of red block in Figure 3) was tested, which varies from 0 to 6. The coverage is fixed to 5.

The procedure of each Monte Carlo trial is as follows: (1) All the reported variations of chromosome 1 and 21 of NCBI36/hg18 were removed, and 10 sequences of length 1 Mbp were extracted. Here, the removed CNVs were retrieved from the database of genomic variants (DGV, http://projects.tcag.ca/variation/), including all the discovered CNVs reported in the literature. Then, a sequence was selected randomly among the 10, and was concatenated with its duplication, yielding the reference genome of length 2 Mbp. This reference genome was also used as the control genome. Since we only introduce one CNV in each genome for efficient comparison, a genome of 2 Mbp is large enough. (2) A CNV with copy number *c* and single copy length *l* was introduced artificially to generate the test genome (see Figure 3, where the copy number varies from 2 to 4). Copy number 2 is assumed to be normal; copy number smaller than 2 (0 and 1) indicates deletion event; and copy number larger than 2 (3 and 6) indicates duplication event. (3) SNPs and indels were introduced. The frequency is 5 SNPs/kbp and 0.5 indels/kbp respectively, and the indels have random length of 1 ∼3 bp. (4) Short reads were sampled on both control and test genome to simulate the short-gun sequencing. In such a case, read counts follow the Poisson distribution with the density parameter proportional to the copy number. To simulate the non-uniform bias, the reads were sampled with a sample probability *p*, which is the product of mapability and GC-content profile. Each read has the length of 36 bp to agree with the Illumina platform. We note that, all the studies in the paper used the data that simulate the Illumina platform but the proposed method can be applied to other NGS platforms with longer read length. (5) The short reads were aligned to the reference genome by using Bowtie [29]. Since a read may align to multiple loci, there are mainly two ways to handle this issue: one way is to report only the uniquely mapped read [13], while the other is to select randomly one among the multiple aligments [22]. These two ways have been discussed in [28,29,55]. In this work, the default setting of Bowtie (similar to MAQ’s default policy [29]) is used such that best alignments with less mismatches are reported. When a read has multiple alignments with the same quality score, a random locus is assigned. (6) Finally, CNV-TV and other CNV detection methods were called. Their outputs, *i.e.*, estimates of both change-point position and copy number, were compared with the ground truth (*i.e.,*parameters used in introducing CNVs into the test genome in Step (2)).

**Figure 4:**
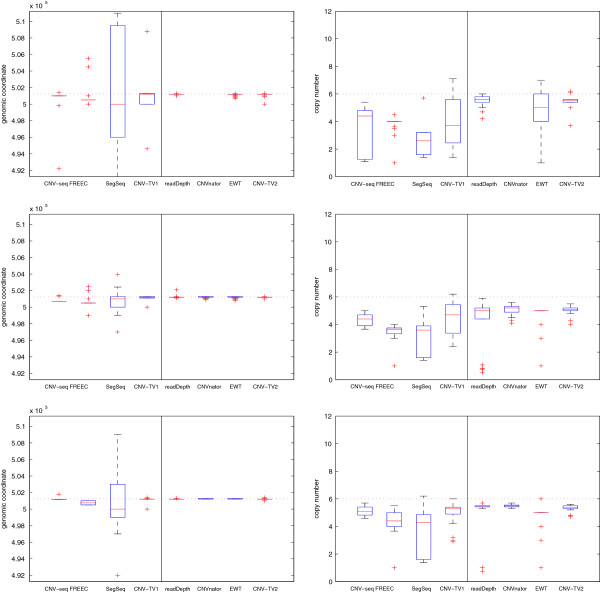
**The box plots of the break point position estimates (first column) and the copy number estimates (second column) of CNVs for different detection methods, and with different single copy lengthes: 1 kbp (first row), 2 kbp (second row) and 6 kbp (third row).** The coverage is fixed to 5, and copy number is fixed to 6. The horizontal red dotted lines indicate the ground truth values; the red solid lines indicate the median values; and the red pluses indicate the outliers. It can be seen that our proposed CNV-TV method gives more robust estimate of both the break point position and copy numbers (*e.g.*, with smaller variance) than other methods for CNVs of different single copy length.

**Figure 5:**
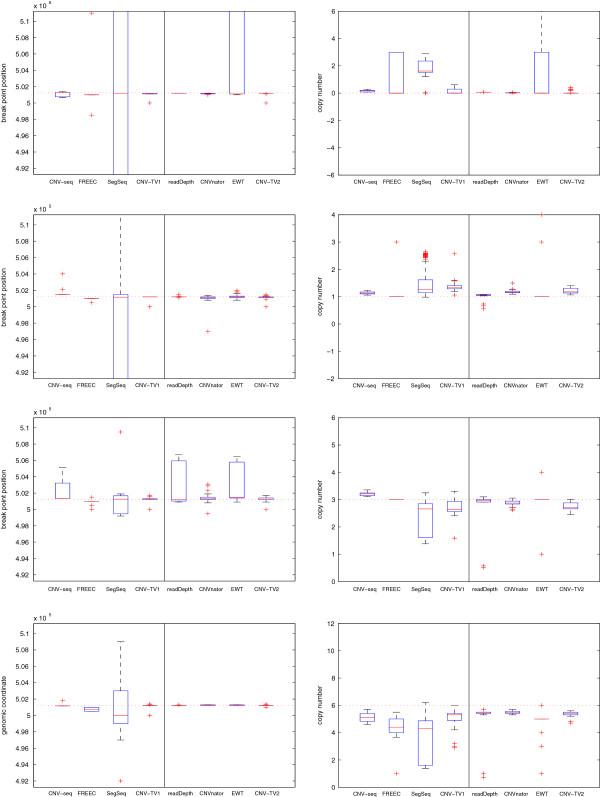
**The box plots of the break point position estimates (first column) and the copy number estimates (second column) of CNVs with different copy number: 0, 1, 3 and 6 (from the first row to the last row).** The coverage is fixed to 5, and the single copy length is fixed to 6 kbp. The horizontal red dotted lines indicate the ground truth values *b*; the red solid lines indicate the median value; and the red pluses indicate outliers. It indicates that our proposed CNV-TV method gives more robust estimates of both the break point position and copy number than other methods for CNVs of different copy numbers.

The false positive rate (FPR, equivalent to 1-specificity) *v.s.* true positive rate (TPR, or sensitivity) of these detection methods are listed in Tables 1 and 2. The FPR is defined as the ratio between the number of false detected CNV loci and that of ground truth normal loci, in the unit of base pair; the TPR is defined as the ratio between the number of true detected CNV loci and that of ground truth CNV loci. The box plots (which includes the minimum, the lower quartile, the median, the upper quartile and the maximum) of the estimates of both the break point locus and copy number are displayed in Figures 4 and 5; the means and standard deviations of the estimation errors are shown in Additional file 1: Tables S1 and S2 respectively. Since CNV-seq, FREEC and SegSeq need control samples, while readDepth, CNVnator and EWT do not, they are displayed in two groups respectively. Correspondingly, ‘CNV-TV1’ indicates the test-control setting, in which the input *x*_*i*_ is the read depth signal ratio between the test and the control sample; ‘CNV-TV2’ indicates the test-only setting. We found that the methods to be compared fail occasionally; for example, CNVnator degenerates when the length of CNV is small (see Table 1); readDepth and CNV-seq fail when the copy number is close to the normal one (*c*=2, see Table 2). However, it can be seen that there are little changes on the estimates with CNV-TV with respect to both the single copy length *l* and the copy number *c*, indicating more robust performance of CNV-TV than that of other methods.

**Table 1:** **The detection FPR/TPR with different single copy length *l***

**l**	**CNV-seq**	**FREEC**	**SegSeq**	**CNV-TV1**	**readDepth**	**CNVnator**	**EWT**	**CNV-TV2**
1e3	4.7e-4/0.97	1.5e-3/1.00	6.7e-3/0.99	2.3e-3/0.97	4.5e-5/0.96	1.7e-6/0.07	2.3e-4/0.99	1.0e-4/0.97
2e3	4.5e-4/0.96	1.4e-3/1.00	5.0e-3/1.00	1.5e-3/0.98	6.5e-5/0.98	1.0e-4/0.96	3.0e-4/0.99	7.7e-5/0.98
6e3	3.5e-4/1.00	9.9e-4/1.00	4.9e-3/0.99	7.9e-4/0.99	3.1e-5/0.99	2.5e-5/0.99	1.3e-4/0.99	6.2e-5/0.99

**Table 2:** **The detection FPR/TPR with different copy number *c***

**c**	**CNV-seq**	**FREEC**	**SegSeq**	**CNV-TV1**	**readDepth**	**CNVnator**	**EWT**	**CNV-TV2**
0	3.4e-4/0.98	2.1e-3/1.00	4.8e-3/0.00	1.5e-3/0.99	4.0e-5/0.99	1.3e-4/0.99	3.4e-4/0.99	2.2e-4/0.99
1	0.0e-0/0.23	5.2e-4/0.99	4.4e-3/0.95	1.4e-3/0.98	3.0e-5/0.30	3.4e-4/0.95	2.5e-4/0.98	4.2e-4/0.98
3	1.4e-5/0.05	7.2e-4/0.97	4.7e-3/0.85	2.9e-3/0.98	1.9e-5/0.06	2.2e-4/0.92	2.8e-4/0.82	4.6e-4/0.99
6	3.5e-4/1.00	9.9e-4/1.00	4.9e-3/0.99	7.9e-4/0.99	3.1e-5/0.99	2.5e-5/0.99	1.3e-4/0.99	6.2e-5/0.99

### Real data processing

To demonstrate the performance of CNV-TV with real data, and compare the quality of detected CNVs with other methods, mapped reads data (BAM files) were downloaded from the 1000 Genomes Project at http://ftp.1000genomes.ebi.ac.uk/. The reads were sequenced from the chromosome 21 of NA19240 (yoruba female) with SLX, Illumina Genome Analyzer. There are 33.4 million reads uniquely aligned to NCBI36/hg18.

**Figure 6:**
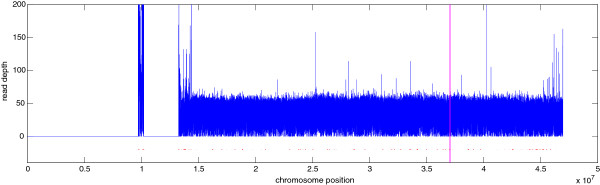
**Chromosome 21 of NA19240.** The blue curve is the read depth signal, the red dots below are detected CNV regions. Zoom in of the region within the two vertical magenta lines is displayed in Figure 1.

Figure 6 shows the read depth signal (blue line) as well as the detected CNV regions (red dots below), and the enlarged view of the region 37.0 ∼37.1 Mbp (region within the two vertical magenta lines) is displayed in Figure 1. The overlaps of CNVs detected by the CNV-TV, and other six methods, as well as those listed in DGV [2], were displayed by an 8-way Venn diagram, whose unit is a block of size 100 bp. Since the 8-way Venn diagram is too complicated to visualize (there are totally 2^8^−1=255 domains), it is tabularized in a binary manner, as shown in Table 3, which only lists the domains with block number greater than 1000. For example, the first column means that there are 31144 blocks that are uniquely detected by SegSeq but are not detected by any other methods or in DGV. Here we used the beta version of DGV, where CNVs can be retrieved by sample, platform, study, *etc.* The option of filter query was ‘external sample id = NA19240, chromosome = 21, assembly = NCBI36/hg18, variant type = CNV’. Table 3 shows that most of the CNVs detected by CNV-TV are consistent with other methods, demonstrating the robustness and reliability of our proposed method. Nevertheless, CNV-TV also reported a small amount of uniquely detected CNVs with length around 1 kbp, *e.g.*, the region at 37.04 Mbp in Figure 1.

**Table 3:** 8-way tabularized Venn diagram of the detected CNVs in the sample NA19240

CNV-seq	0	1	1	0	1	0	0
FREEC	0	1	1	0	1	0	0
readDepth	0	1	1	0	1	1	0
CNVnator	0	1	1	1	1	0	1
SegSeq	1	0	1	0	1	0	0
EWT	0	1	1	0	1	0	1
CNV-TV	0	1	1	0	1	0	0
DGV	0	0	0	0	1	0	0
Block							
numbers	31144	2637	2535	2213	1458	1331	1065

‘1’ encodes that a CNV can be detected with a method while ‘0’ encodes a failure (*e.g.* the first column means that there are 31144 blocks that are detected by SegSeq, but can not be detected by any other methods or included in the DGV).

The *F*-score [56] measures the overlap quality between two sections. It takes values between 0 and 1. A low score indicates poor quality overlap while a high score indicates good quality overlap. The *F*-score is calculated as $F=2\frac{\text{PR}}{P+R}$, where *P* is the precision (percent of detected CNVs that overlap with the ground truth CNVs from DGV) and *R* is the recall (percent of the ground truth CNVs which overlap with the detected CNVs). Table 4 lists the top 10 *F*-scores of each method, and the corresponding *P* and *R* are listed in the Additional file 1 (Tables S3 and S4). It can be seen that the CNV-TV method can provide CNVs with higher *F*-scores, indicating better quality compared with other methods.

**Table 4:** ***F*-scores of top 10 CNVs detected by each method from the sample NA19240**

**CNVs**	**1**	**2**	**3**	**4**	**5**	**6**	**7**	**8**	**9**	**10**
CNV-seq	0.74	0.73	0.64	0.62	0.53	0.42	0.40	0.30	0.05	0.04
FREEC	0.88	0.83	0.65	0.64	0.63	0.63	0.53	0.53	0.48	0.43
readDepth	0.81	0.80	0.79	0.75	0.74	0.71	0.71	0.64	0.62	0.57
CNVnator	0.92	0.90	0.88	0.84	0.82	0.82	0.79	0.78	0.77	0.73
SegSeq	0.93	0.91	0.89	0.59	0.57	0.55	0.48	0.45	0.45	0.45
EWT	0.93	0.92	0.78	0.77	0.73	0.64	0.58	0.56	0.41	0.24
CNV-TV	0.92	0.86	0.81	0.79	0.78	0.74	0.74	0.69	0.58	0.57

Five more sequence data were also processed, which were sampled from chromosome 21 of a CEU trio of European ancestry: NA12878 the daughter, NA12891 the father and NA12892 the mother, a Yoruba Nigerian female NA19238, and a male NA19239. The 8-way Venn diagram analysis shows that on average 98.7% of CNVs detected by the CNV-TV overlap with at least one CNV by other method, or DGV. This number for CNV-seq is 97.8%, FREEC 97.1%, readDepth 89.5%, CNVnator 85.2%, SegSeq 22.4%, EWT 78.3%, respectively.

Table 5 summarizes the average distributions of *F*-score of the detected CNVs of each method over the six sequence data. Each detected CNV is cataloged into 10 classes (0∼0.1,0.1∼0.2,…,0.9∼1) according to its *F*-score. It is shown that the CNV-TV reports less low quality detections (*F*-score is lower than 0.1) and more high quality detections (*F*-score is greater than 0.5), indicating its robust performance.

**Table 5:** **Average distribution (in percentage) of *F*-scores of detected CNVs in the real data processing**

***F*-score**	**0*. *0∼ 0*.*1**	**0*. *1∼ 0*.*2**	**0*. *2∼ 0*.*3**	**0*. *3∼ 0*.*4**	**0*. *4∼ 0*.*5**	**0*. *5∼ 0*.*6**	**0*. *6∼ 0*.*7**	**0*. *7∼ 0*.*8**	**0*. *8∼ 0*.*9**	**0*. *9∼ 1*.*0**
CNV-seq	86.27	2.88	2.15	1.62	1.79	0.95	1.85	1.79	0.71	0.00
FREEC	85.25	4.28	2.43	1.79	1.51	1.19	1.48	0.90	1.17	0.00
readDepth	94.47	0.91	0.53	0.99	0.65	0.42	0.45	1.18	0.38	0.00
CNVnator	89.72	2.56	0.97	0.69	1.18	0.94	0.93	1.31	0.93	0.76
SegSeq	89.59	3.19	2.10	1.03	1.60	1.03	0.45	0.20	0.35	0.41
EWT	96.12	0.67	0.48	0.49	0.38	0.31	0.28	0.75	0.19	0.32
CNV-TV	83.71	3.13	2.20	1.39	1.74	2.57	0.74	2.43	1.57	0.49

The experiments were carried out on a desktop computer with a dual-core 2.8 GHz x86 64 bit processor, 6 GB memory and openSUSE 11.3. CNV-TV finished the processing in 112.2 seconds with peak memory usage of 383.4 Mega bytes. The computation time and memory usage of CNV-seq, FREEC, readDepth, CNVnator, SegSeq and EWT are 251.5, 319.6, 134.8, 162.6, 248.8 and 268.9 seconds, 27.1, 7.1, 1060.1, 101.9, 3508.4, and 156.6 Mega bytes, respectively. This shows that the CNV-TV is the fastest in computation with reasonable memory usage.

## Conclusion and discussion

In this paper, we proposed the CNV-TV method based on total variation penalized least squares optimization, in order to detect copy number variation from next generation sequencing data. The proposed method assumes that the read depth signal is piecewise constant, and the plateaus and basins of the read depth signal correspond to duplications and deletions respectively. Here three major points should be highlighted: (1) The proposed CNV-TV method is quite automatic. We use the SIC to determine the tuning of the penalty parameter for the control of the tradeoff between TPR and FPR, which is often cumbersome to do. (2) The method can be applied to either matched pair data or single data adjusted for technical factors such as the GC-content correction. (3) The method has better robustness, more reliability, and higher detection resolution. We compared the CNV-TV method with six other CNV detection methods. The simulation studies show that the detection performance of CNV-TV in terms of break point position and copy number estimation are more robust compared with six other methods under a set of parameters (*e.g.*, different single copy lengths and copy numbers). The test on real data processing demonstrates that CNV-TV gives higher resolution to detect CNVs of smaller size. In addition, the method can detect CNVs with higher *F*-scores, showing better quality compared with other methods.

The simulation results (Tables 1, 2, Additional file 1: Tables S1, and S2) show that CNV-TV gives slightly lower FPR and estimation error than those of FREEC when the single copy length is 6 kbp, and the copy number is 0. Real data processing results (Tables 4 and 5) indicate that CNV-TV can detect CNVs with higher *F*-score compared with FREEC. However, both simulation and real data processing results show that the overall performances of FREEC and CNV-TV are similar. Since both of them formulate the CNV detection problem as a change-point detection based on sparse representation, and use the LASSO to solve the problem. Therefore it is worthwhile to show their differences and connections. The first is that the two methods use different models. FREEC uses the method proposed by Harchaoui and Lévy-Leduc [40], in which the matrix ***A*** in Eq. (4) is an *n*×*n* lower triangular matrix with nonzero elements equal to one; in our CNV-TV method, the ***A*** matrix is an *n*×(*n*−1) triangular matrix. These two matrices are closely related, but with the difference up to a projection procedure implied in Eq. (5). The second lies in the method to determine the number of change-points. FREEC uses the LASSO to select a set of candidate change-points, and the number of the change points is up-bounded by a predefined value *K*_*max*_. Then it uses the reduced dynamic programming (rDP) to determine the best number of change-points among the candidates. CNV-TV uses the SIC to determine the number of change-points, which takes the complexity of the model into account. The computational complexity of rDP and SIC are $O(K_{\text{max}}^{3})$ and $O(K_{\text{max}})$ respectively. When *K*_*max*_ is large, especially being true for whole genomic data analysis, CNV-TV can save computation significantly.

Our proposed CNV-TV is based on DOC profile and therefore we make the comparison currently with those methods also based on DOC. Because large events can be detected with DOC profile while small events can be detected with PEM signature, these two signatures provide complementary information. A good strategy is to combine these two signatures as described in methods [16,17,57]. These methods use the DOC signature to detect the coarse region of CNV, and then estimate the fine locus of the break points with PEM signature. In addition, the analysis of tandem duplication regions is also challenging since one read may have multiple alignment loci. A simple way to alleviate this issue is to randomly assign a locus. Another way is to increase the read length, which can decrease the frequency of multiple alignment. He *et al.*[58] proposed to use the discordant read pairs and unmapped reads that span on the break points to detect CNVs, and the precision of detected CNV break points can reach at base pair level. So our future work will consider the incorporation of multiple signatures into algorithm design, which could further improve CNV detection accuracy.

## Competing interests

The authors declare that they have no competing interests.

## Authors’ contributions

JD, J-GZ, Y-PW and H-WD designed this study. JD and J-GZ wrote the code for the comparative study. JD wrote the manuscript, J-G Zhang and Y-PW revised the manuscript. All have read the manuscript and approved the final version.

## Supplementary Material

Additional file 1 Appendix. Click here for file
