# Supplementary material for: CNV-TV: A robust method to discover copy number variation from short sequencing reads
Source: BMC Bioinformatics. 2013 May 2;14:150. doi: 10.1186/1471-2105-14-150 (PMC3679874; doi:10.1186/1471-2105-14-150)
Supplement: Additional file 1 — Appendix. [file 1471-2105-14-150-S1.docx]

Table S1: The mean and standard deviations of estimation error of break point position.

|  | CNV-seq | FREEC | SegSeq | CNV-TV1 | readDepth | CNVnator | EWT | CNV-TV2 |
| --- | --- | --- | --- | --- | --- | --- | --- | --- |
| *l*=1 kbp, *c*=6 | 365±1.4e3 | 708±930 | 700±1.8e3 | 238±637 | 30±12 | NA | 35±16 | 63±17 |
| *l*=2 kbp, *c*=6 | 449±218 | 793±752 | 884±552 | 123±355 | 29±92 | 2±91 | 83±82 | 32±27 |
| *l*=6 kbp, *c*=6 | 100±138 | 479±251 | 1.2e3±1.4e3 | 163±515 | 28±22 | 2±48 | 31±14 | 16±38 |
| *l*=6 kbp, *c*=0 | 116±270 | 359±548 | 417±9.1e3 | 235±439 | 33±0 | 113±51 | 5.1e3±5.1e3 | 48±121 |
| *l*=6 kbp, *c*=1 | 613±838 | 274±136 | 194±5.3e3 | 118±311 | 25±80 | 177±442 | 30±194 | 255±484 |
| *l*=6 kbp, *c*=3 | 1.3e3±1.6e3 | 269±178 | 561±1.1e3 | 35±261 | 1.6e3±2.2e3 | 79±404 | 1.7e3±2.1e3 | 65±409 |

Table S2: The mean and standard deviations of estimation error of copy number.

|  | CNV-seq | FREEC | SegSeq | CNV-TV1 | readDepth | CNVnator | EWT | CNV-TV2 |
| --- | --- | --- | --- | --- | --- | --- | --- | --- |
| *l*=1 kbp, *c*=6 | 2.43±1.45 | 2.50±0.24 | 3.47±1.07 | 2.00±1.38 | 0.90±0.70 | NA | 1.35±0.94 | 0.54±0.37 |
| *l*=2 kbp, *c*=6 | 1.61±0.22 | 2.3±0.51 | 2.86±1.07 | 1.03±1.06 | 1.23±1.08 | 0.85±0.22 | 1.30±0.79 | 0.91±0.23 |
| *l*=6 kbp, *c*=6 | 0.87±0.19 | 1.56±0.53 | 2.46±1.56 | 0.83±0.20 | 0.82±0.78 | 0.50±0.08 | 1.10±0.93 | 0.61±0.13 |
| *l*=6 kbp, *c*=0 | 0.15±0.06 | 1.12±1.41 | 1.73±0.70 | 0.05±0.12 | 0.06±0.01 | 0.02±0.01 | 1.27±2.08 | 0.02±0.12 |
| *l*=6 kbp, *c*=1 | 0.14±0.05 | 0.15±0.53 | 0.49±0.46 | 0.26±0.39 | 0.02±0.13 | 0.17±0.05 | 0.36±0.89 | 0.22±0.10 |
| *l*=6 kbp, *c*=3 | 0.21±0.08 | 0.00±0.00 | 0.63±0.59 | 0.46±0.48 | 0.46±0.99 | 0.12±0.08 | 0.03±0.30 | 0.25±0.13 |

Table S3: P-scores of top 10 CNVs detected by each method from the sample NA19240.

| method\CNVs | 1 | 2 | 3 | 4 | 5 | 6 | 7 | 8 | 9 | 10 |
| --- | --- | --- | --- | --- | --- | --- | --- | --- | --- | --- |
| CNV-seq | 0.844892 | 1 | 0.812483 | 1 | 1 | 0.269652 | 0.251343 | 1 | 1 | 1 |
| FREEC | 1 | 0.727136 | 0.526987 | 0.77975 | 1 | 1 | 0.76012 | 1 | 0.315592 | 0.810274 |
| readDepth | 0.7 | 0.9225 | 1 | 1 | 0.588333 | 0.997 | 0.785 | 0.67 | 1 | 1 |
| CNVnator | 0.9775 | 0.817273 | 0.778571 | 0.723684 | 1 | 0.715714 | 0.925 | 0.633333 | 0.816667 | 0.574444 |
| SegSeq | 1 | 0.841869 | 0.809133 | 0.415202 | 0.426527 | 0.575028 | 1 | 0.294023 | 0.291202 | 0.339576 |
| EWT | 0.9975 | 0.855714 | 1 | 0.97 | 1 | 1 | 1 | 0.392222 | 0.26 | 1 |
| CNV-TV | 0.937778 | 1 | 0.7172 | 0.646923 | 0.74 | 0.61 | 0.589655 | 1 | 0.59875 | 0.399615 |

Table S4: R-scores of top 10 CNVs detected by each method from the sample NA19240.

| method\CNVs | 1 | 2 | 3 | 4 | 5 | 6 | 7 | 8 | 9 | 10 |
| --- | --- | --- | --- | --- | --- | --- | --- | --- | --- | --- |
| CNV-seq | 0.662573 | 0.572258 | 0.522149 | 0.447456 | 0.364966 | 1 | 1 | 0.178796 | 0.025265 | 0.021029 |
| FREEC | 0.780117 | 0.976838 | 0.83591 | 0.543948 | 0.458658 | 0.455757 | 0.411526 | 0.356122 | 1 | 0.288557 |
| readDepth | 0.965517 | 0.713733 | 0.653595 | 0.600601 | 1 | 0.554505 | 0.640816 | 0.609091 | 0.452489 | 0.395257 |
| CNVnator | 0.861233 | 1 | 1 | 1 | 0.700701 | 0.963462 | 0.694618 | 1 | 0.722714 | 1 |
| SegSeq | 0.861931 | 1 | 1 | 1 | 0.841019 | 0.53331 | 0.315195 | 1 | 1 | 0.657005 |
| EWT | 0.875 | 0.999444 | 0.641026 | 0.640969 | 0.57843 | 0.475624 | 0.412414 | 1 | 1 | 0.139361 |
| CNV-TV | 0.904609 | 0.747763 | 0.938089 | 1 | 0.814679 | 0.954481 | 1 | 0.529187 | 0.571599 | 1 |
